# Redefining the high‐grade B cell lymphoma with double/triple rearrangements of MYC and BCL2/BCL6 genes. Learning from a case report

**DOI:** 10.1002/jha2.310

**Published:** 2021-11-09

**Authors:** Socorro María Rodríguez‐Pinilla, Rocío Nieves Salgado, Cristina Chamizo, Carlos Santonja, Peter Stewart, Nerea Carvajal, Neil McCafferty, Rebeca Manso, Daniel Morillo, Miguel Ángel Piris, David González de Castro

**Affiliations:** ^1^ Pathology Department Hospital Universitario Fundación Jiménez Díaz (FJD) Madrid Spain; ^2^ Cytogenetic Department Hospital Universitario Fundación Jiménez Díaz (FJD) Madrid Spain; ^3^ Patrick G Johnston Centre for Cancer Research Queen's University Belfast Belfast UK; ^4^ Haematology Department Hospital Universitario Fundación Jiménez Díaz Madrid Spain

**Keywords:** B‐cell lymphomas with double or triple hits, fluorescence in situ hybridization, follicular lymphoma, *MYC*, *PVT1*

## Abstract

We report a patient initially diagnosed with a triple hit high‐grade B cell lymphoma (HGBL‐TH), in which further morphologic, immunohistochemical, and next‐generation sequencing studies of subsequent specimens disclosed it to be a germinal center diffuse large B cell lymphoma (GC‐DLBCL) with *BCL2/BCL6* gene translocations, *PVT1*‐deletion, and gain of *MYC* genes evolving from a previous follicular lymphoma. However, fluorescence in situ hybridization (FISH) studies with the break‐apart probe for MYC gene showed a fusion and two separated signals (red and green, respectively) leading to the interpretation of MYC gene translocation and a false diagnosis of a TH‐lymphoma, according to the recent WHO classification. Nevertheless, *PVT1* deletion plus *MYC* gain/amplification has been described as a cause of the double‐hi transcription profile. These data highlight the need for new criteria to identify these highly aggressive lymphomas.

High‐grade B‐cell lymphomas with double or triple hits (*MYC* plus *BCL2* and/or *BCL6* genes rearrangements) (HGBL‐DH/TH) are usually highly aggressive lymphomas that respond poorly to conventional chemotherapeutic regimes [1]. Nowadays, the fluorescence in situ hybridization (FISH) study with break‐apart probes for *MYC* is the standard diagnostic method in pathology laboratories, although a relatively high rate of false‐negative results, is commonplace [1, 2, 3]. Moreover, there is no consensus on the FISH pattern required to classify a case as translocated: even though single yellow and separate red and green dots are typically found in a translocated case; other combinations are also regarded as positive by most experts [4].

We report a patient initially diagnosed with an HGBL‐TH, in which further morphologic, immunohistochemical, and next‐generation sequencing (NGS) studies of subsequent specimens disclosed it to be a germinal center diffuse large B cell lymphoma (GC‐DLBCL) with *BCL2/BCL6* gene translocations, *PVT1*‐deletion, and gain of *MYC* genes evolving from a previous follicular lymphoma (FL). The patient suffered frequent recurrences, with no response to several lines of chemotherapy. After Chimeric Antigen Receptor (CAR) T‐cell therapy, he is alive and free of disease. According to the current 2016 WHO classification, this case would not meet diagnostic criteria for HGBL‐DH/TH [1]. Recently, gene expression studies have identified a subgroup of aggressive lymphomas with double‐hit (DH) signatures which might benefit from intensive chemotherapeutic regimes (SHA and Ennish). Interestingly, only half of the cases identified by the so‐called molecular high‐grade signature (MHG) (Sha) or the DH‐signature (Ennish), respectively, represented cases with double *MYC/BCL2* rearrangements using conventional FISH break‐apart probes [3]. Among the other cases showing an MHG /DH signature, some disclosed *PVT1* gene deletion with further gain/amplification of the *MYC* gene [3]. These data reveal two aspects that need to be addressed: on the one hand, reliable guidelines for FISH interpretation should be set. On the other hand, tools superior to FISH studies are needed to identify these highly aggressive lymphomas.

A 57‐year‐old man with no previous medical history presented with weight loss, abdominal discomfort, and without fever or B‐symptoms. Physical examination and imaging studies revealed generalized lymphadenopathy. A supraclavicular lymph node was excised (A). Based on morphology, immunophenotype (Supplementary Table S1), and FISH studies, a diagnosis of HGBC‐DH/TH was made (Figures 1 and 2 and Supplementary Figures S1–S3). Twenty days later, the patient complained of intense abdominal pain and image studies revealed an increase in the number and size of the previously detected lymph nodes, as well as signs of perforated hollow viscus with abundant gas and fluid in the peritoneal cavity. An emergency resection of a small bowel segment was performed. The specimen showed full‐thickness involvement by a 10‐cm in length bosselated and ulcerated mass, with numerous accompanying enlarged lymph nodes. A diagnosis of HGBC‐DH/TH was made in the intestinal mass (Figures 1 and 2; Supplementary Table S2, Supplementary Figure S4) (B3); moreover, in situ follicular neoplasia (ISFN) (B1) was detected in the adjacent, uninvolved mucosa (Figures 1 and 2, Supplementary Table S2, Supplementary Figure S5), and a well‐established FL evolving into an HGBC‐DH was seen in lymph nodes of the perivisceral fat (Figures 1 and 2, Supplementary Table S2, Supplementary Figure S6) (B2). FISH studies were done in all samples while conventional cytogenetic studies were performed in sample A (Supplementary Figures S1 and S2). NGS and RNA sequencing studies were done in samples A, B2, and B3 (see the Supporting Information and Methods section, Supplementary Table S2; Figure 2).

**FIGURE 1:**
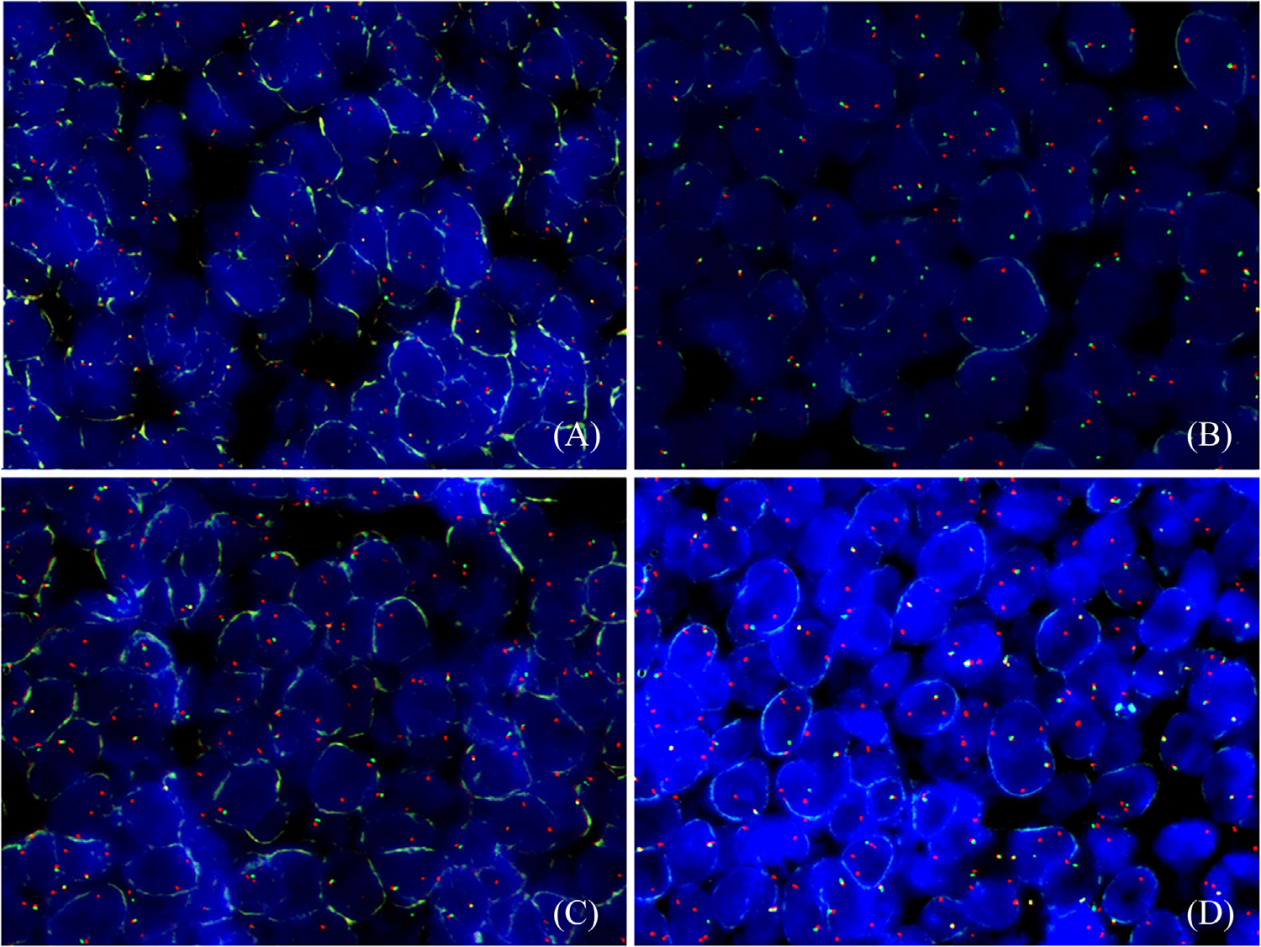
FISH studies of the *MYC* gene in the four different samples of the patient. The Vysis LSI MYC break‐apart rearrangement probe kit from Abbott was used (ref: 01N63‐020). (A) In situ neoplasm in the intestine (sample B1), 2F are seen. (B) FI‐DLBCL sample (sample B2), 1R, 1G, 1F is seen. (C) DLBCL‐I sample (sample B3), 1F, 1R is seen, and (D) DLBCL‐LN sample (sample A), 1F, 2R are seen. F = fusion, yellow dot; R = red dot; G = green dot

**FIGURE 2:**
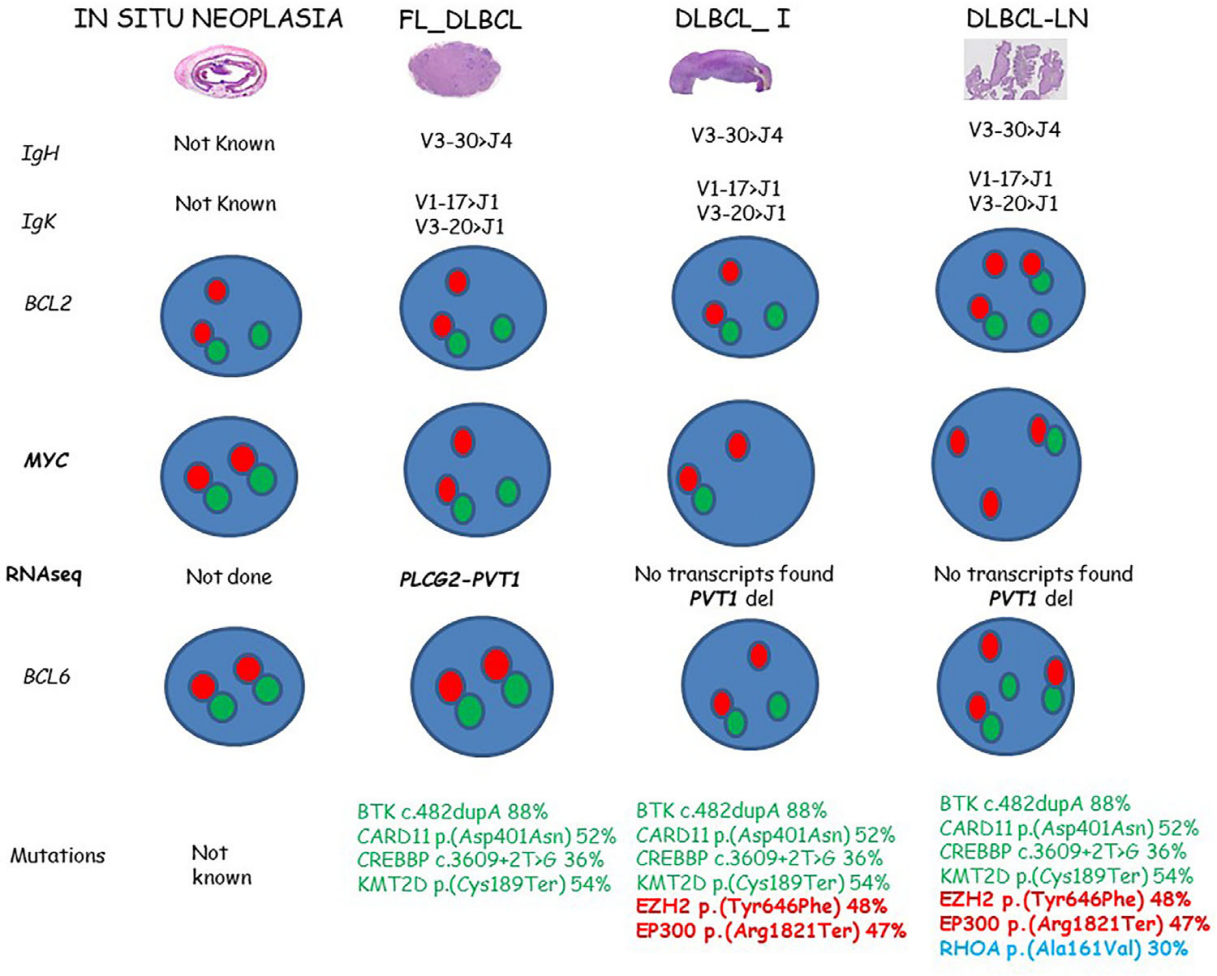
Molecular mechanisms implicated in the transformation of FL to a DLBCL. Clonal multistep progress of acquisition of genetic alterations is proposed. Different molecular alterations in the same patient at different site biopsies at the same time of disease were found

The patient received three cycles of DA‐EPOCH‐R + HD‐methotrexate and achieved complete remission (CR), but suffered recurrence 5 months later and R‐ESHAP was administered, with no response. After the progression of the disease, treatment with R‐GEMOX was likewise unsuccessful. In February 2020, he was selected for CART therapy, achieving CR. He remains free of disease after 10 months of follow‐up.

Molecular mechanisms implicated in the transformation of FL to DLBCL are heterogeneous, and most studies suggest a divergent evolution from a common progenitor cell. In the present case*, BCL2/IGH* rearrangement was seen (specifically to *IGHJ6*) by NGS, in all studied samples. Moreover, the three samples analyzed shared the same *IgH* and *IgK* rearrangement confirming the clonal relationship between them. Furthermore, both the somatic mutation repertoire and the pattern of FISH alterations of *BCL2*, *BCL6*, and *MYC* genes suggest the development of the DLBCL by a linear evolution from an FL dominant clone (Figures 1 and 2; Supplementary Table S2).

Alterations in *BTK*, *CARD11*, *CREBBP*, and *KMT2D* genes were shared by the three samples studied. It is well known that mutations in genes involved in epigenetic regulation, chromatin modification, *JAK‐STAT*‐pathway, and *B‐cell‐receptor (BCR)/NF‐κB* signaling genes dominate the FL landscape pathogenesis [5]. *EZH2* and *EP300* mutations were shared by both samples diagnosed as DLBCL (A and B3), so it can be assumed that they took place as later hits. Interestingly, as a final alteration *RHOA* gene mutation was exclusively found in sample A.

RNA sequencing studies identified a fusion transcript involving *PLCG2* and *PVT1* genes in sample B2 (Figure 2 and Supplementary Table S2) while no transcripts were found in either sample A or sample B3. Interestingly, this transcript was just found in the only sample with a conventional pattern of *MYC* gene rearrangement (1F, 1R, and 1G dots). Interestingly, the study of the CHIMERIC nucleotidic‐sequence by BLAST allows us to realize that *PLCG2* is joined to *PVT1* following the 5′ to the 3′ way of enlargement and that this chimeric does not include the *MYC* gene sequence. The break‐apart probe for *MYC* marks in red the 5′end of the sequence of the gene and green the 3′ end of the sequence. *PVT1* is located close to the 3′‐end region of the *MYC* gene. In sample B2, *PVT1* but not *MYC* is the truly translocated gene. Furthermore, in samples B3 and A, one fusion and either one or two isolated red signals were found, respectively. Moreover, the transcript was not found and the green dot from the *MYC* break‐apart probe was not identified in either B3 and A samples, suggesting deletion of the *PVT1* gene in both samples and further gain of *MYC* (red dot) in sample A. This data highlight the lack of sensitivity of these probes to identify *MYC* gene rearrangements. Moreover, in our hands, 1F and 1R dot signals were a consequence of *PVT1* gene deletion, and in contrast to what Tang et al. [4] described it is not indicative of *MYC* gene translocation. These data highlight the need for consensus guidelines of interpretation of *MYC* FISH studies (Figure 1).

*PVT1* represents a long non‐coding RNA locus that has been identified as a candidate oncogene. *PVT1* deletion plus *MYC* gain/amplification has been described as a cause of DH transcription profile. Moreover, the loss of a translocation with further amplification of the gene is an oncogenic process previously reported in tumors to maintain the overexpression of the implicated protein. Interestingly, genetic lesions deregulating *MYC*, namely chromosomal translocations, copy number gains/amplification, and point mutations are common findings in DLBCL transformed from FL.

Based on FISH studies *BCL6* was translocated, but—according to NGS studies—not to *IgH, IgK*, or *IgL*. *BCL6* translocation in our case was exclusively found on samples A and B3 but not in samples B1/B2 suggesting its development after *PLCG2‐PTV1* rearrangement.

No material from the ISFN could be obtained. There is discussion in the literature regarding the ability of ISFN to evolve into full‐blown FL [6]. Here, differential diagnosis to a duodenal‐type follicular lymphoma (DTFL) which had transformed into a DLBCL should be ruled out. Interestingly, a clonal relationship between ISFN and DTFL has been reported [7].

In summary, we propose that this DLBCL developed from a previous FL that, in turn, had probably evolved from an ISFN. Clonal multistep progress of acquisition of genetic alterations is proposed (Supplementary Figure S4). These data are consistent with previous studies that suggest that FL is a heterogeneous disease in which different molecular alterations in the same patient at different site biopsies at the same time of disease can be found in [8].

In retrospect, this case did not meet the criteria for a diagnosis of HGBL‐DH/TH of the current WHO classification. Nevertheless, this patient's lymphoma behaved aggressively and required CART therapy. It is our view that better tools, probably related to RNA expression, to identify these highly aggressive lymphoma patients are needed.

## CONFLICTS OF INTEREST

Dr. Piris is sponsored by TAKEDA. The other authors have no conflicts to declare.

## Supporting information

SUPPORTING INFORMATION

SUPPORTING INFORMATION

SUPPORTING INFORMATION

SUPPORTING INFORMATION

SUPPORTING INFORMATION

SUPPORTING INFORMATION

SUPPORTING INFORMATION

SUPPORTING INFORMATION
