# Supplementary figures and images for: Redefining the high‐grade B cell lymphoma with double/triple rearrangements of MYC and BCL2/BCL6 genes. Learning from a case report
Source: EJHaem. 2021 Nov 9;3(1):171–4. doi: 10.1002/jha2.310 (PMC9175839; doi:10.1002/jha2.310)

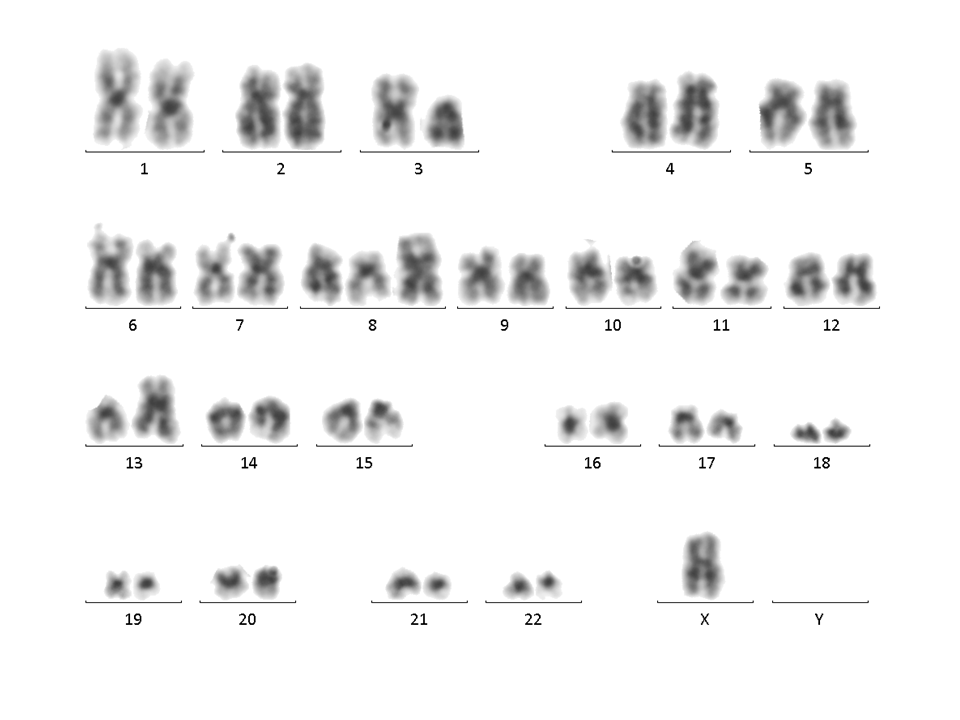

Supplement: Supplementary file 1 — SUPPORTING INFORMATION [file JHA2-3-171-s004.tif]

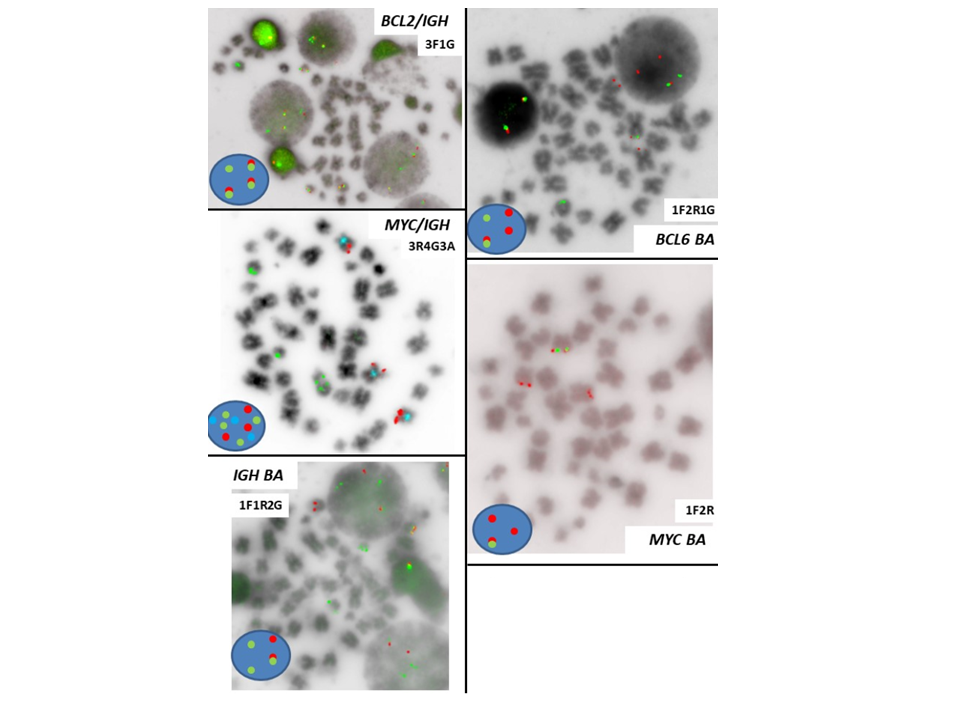

Supplement: Supplementary file 2 — SUPPORTING INFORMATION [file JHA2-3-171-s006.TIF]

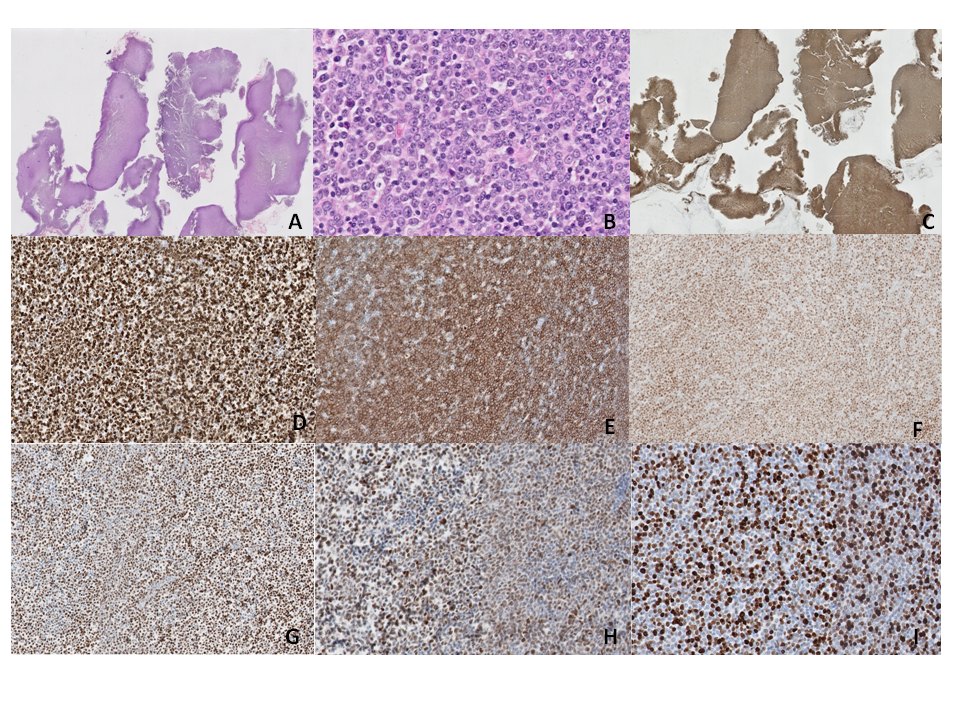

Supplement: Supplementary file 3 — SUPPORTING INFORMATION [file JHA2-3-171-s007.tif]

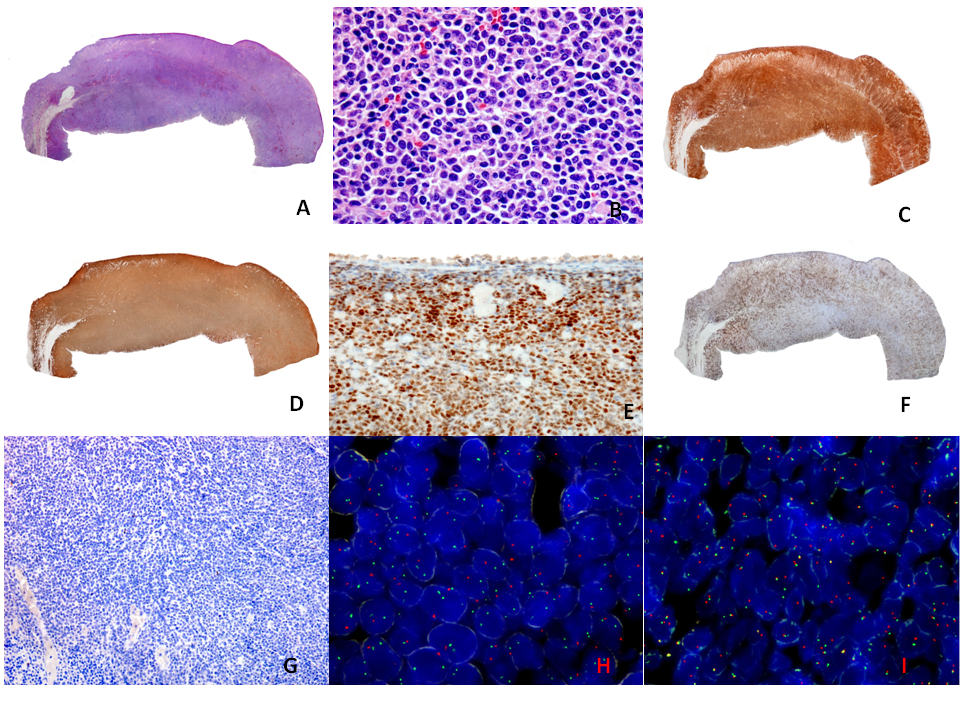

Supplement: Supplementary file 4 — SUPPORTING INFORMATION [file JHA2-3-171-s001.tif]

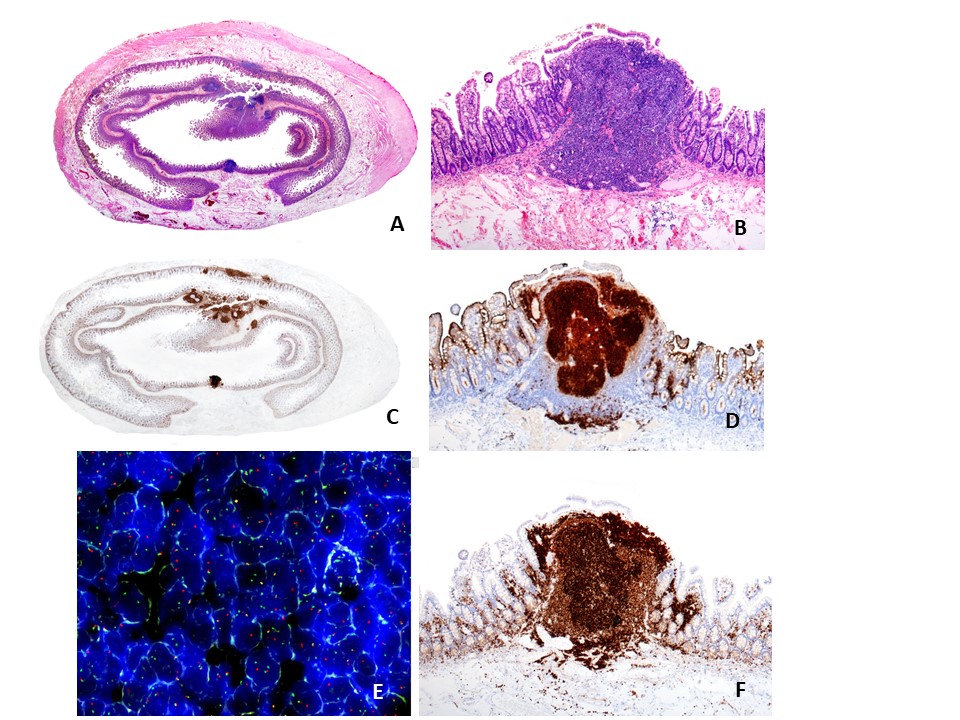

Supplement: Supplementary file 5 — SUPPORTING INFORMATION [file JHA2-3-171-s002.jpg]

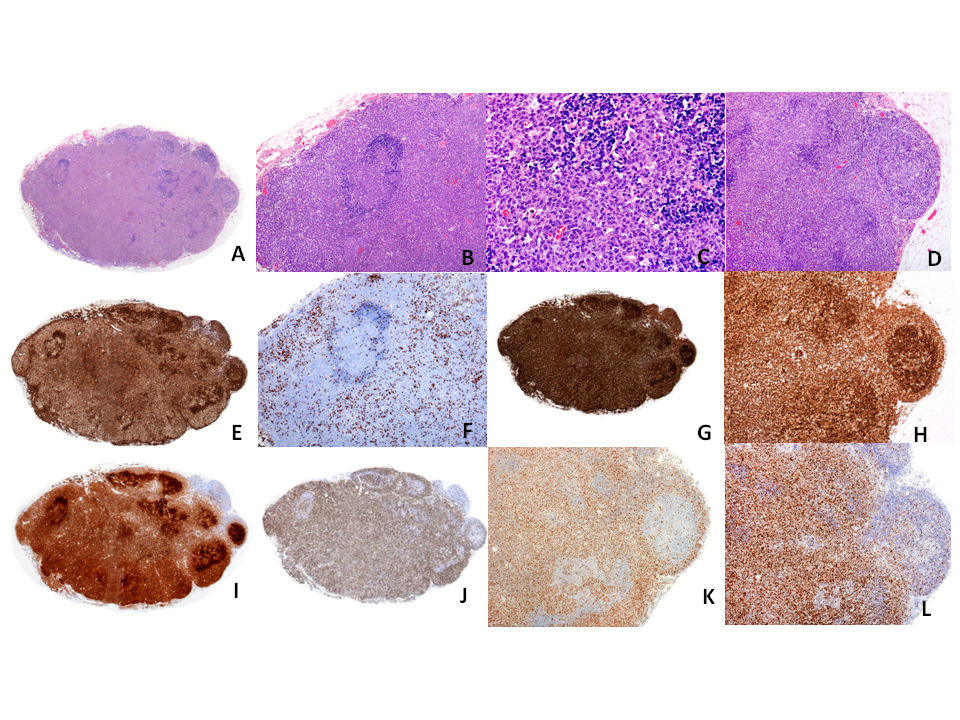

Supplement: Supplementary file 6 — SUPPORTING INFORMATION [file JHA2-3-171-s005.tif]
