# Supplementary material for: Redefining the high‐grade B cell lymphoma with double/triple rearrangements of MYC and BCL2/BCL6 genes. Learning from a case report
Source: EJHaem. 2021 Nov 9;3(1):171–4. doi: 10.1002/jha2.310 (PMC9175839; doi:10.1002/jha2.310)
Supplement: Supplementary file 7 — SUPPORTING INFORMATION [file JHA2-3-171-s003.docx]

**Material and Methods:**

**Immunohistochemistry and in situ hybridization.**

Paraffin sections of 3 to 4 μm in thickness of the biopsy material used for histologic examination were stained with a panel of 18 antibodies and were investigated by in situ hybridization (ISH) for EBV (EBER). See Supplementary Table 1.

**FISH Technique.**

FISH technique was performed in formalin-fixed, paraffin-embedded (FFPE) sections. Briefly, 5 μm-thick formalin-fixed, paraffin-embedded (FFPE) sections were deparaffinized, treated with saline sodium citrate and digested in pepsin solution. In addition, FISH was also carried out in interphase and metaphase cells from lymph node cell culture.

FISH probes used and the expected patterns are summarized in Supplementary Table 2. Probe mixture was prepared according to the manufacturer’s instructions and an appropriate volume was added to each slide. Target DNA and probes were codenatured at 74˚C for 5 minutes and incubated at 37˚C overnight in a humidified hybridization chamber (Thermo-Brite, Abbott Molecular Inc.). Post-hybridization washes were performed in NP40 0.3%/2×SSC (pH 7.0) at 75˚C for 2 minutes. Finally, the slides were air dried and counterstained with DAPI (40, 6-diamidino-2-phenylindole) diluted in Vectashield (Vector, Burlingame, CA, USA). Signal counting was done for each single case over 12 more representative areas.

**Cytogenetics**

Lymph node sample was collected and processed after 24 hours unstimulated culture following standard procedures. G-banding staining was performed using Leishman stain, and the karyotype was reported following the International System for Human Cytogenetic Nomenclature 2016 recommendation. A total of 20 metaphase nuclei were scored.

**DNA and RNA extraction.**

We extracted genomic DNA (gDNA) from FFPE tumour samples using a DNA Sample Preparation kit (Roche) and RNA was extracted using the RNeasy FFPE kit (Qiagen), in accordance with the manufacturer’s protocol. DNA and RNA were quantified with Qubit® (Invitrogen, Carlsbad, CA, USA).

**Next generation sequencing (NGS):**

Next generation sequencing was performed using a customized NGS DNA capture panel and sequenced on a NextSeq 500 (1).

**Trusight RNA Pan-Cancer panel.** RNA was extracted and quantified. The library was prepared with Illumina’s TruSight RNA Pan-Cancer Panel. The sequencing was performed on Miseq Reagent Kit V3 (paired-end, 2x76) on a MiSeq instrument (Illumina, San Diego, CA, USA), as described in the manufacturer’s protocol. FastQ files were generated by BaseSpace Onsite and analyzed further by RNA alignment (Illumina, San Diego, CA, USA). The Trusight RNA Pan-Cancer panel targets 1385 cancer genes and covers 21043 exonic regions with 57010 probes.

**Supplementary Tables:**

**Supplementary Table 1.** Panel of antibodies studied in this study.

| **Antibody** | **Clone** | **Source** | **Dilution** | **pH** |
| --- | --- | --- | --- | --- |
| BCL-6 FLEX | PG-B6p | DAKO | RTU | High |
| BCL-2 FLEX | 124 | DAKO | RTU | High |
| CD20cy | L26 | DAKO | RTU | High |
| CD3-L FLEX | Polyclonal | DAKO | RTU | Low |
| CD10-L FLEX | 56C6 | DAKO | RTU | Low |
| CD23-L FLEX | DAK-23 | DAKO | RTU | Low |
| CYCLIN-D1 | EP-12 | DAKO | RTU | High |
| KAPPA | Polyclonal | DAKO | RTU | High |
| LAMBDA | Polyclonal | DAKO | RTU | High |
| MUM1 | MUM1p | DAKO | RTU | High |
| CD30 | Ber-H2 | DAKO | RTU | Low |
| Ki-67 FLEX | MIB-1 | DAKO | RTU | Low |
| C-MYC | Y69 | Roche | RTU | CC1 |
| P53 | DO-7 | DAKO | RTU | CC1 |
| TDT | Poly | DAKO | RTU | High |
| Histosonda EBER | DNP probe | Roche | RTU | Protease |
| SOX11 | MRQ-58 | Roche | 1/500 | CC1 |
| CD5 | 4C7 | DAKO | RTU |  |
| AID | JUA51E | CNIO | RTU |  |

**Supplementary Table 2.** Panel of FISH probes studied in this study

| **FISH Probe** | **Type** | **Target** | **Expected patterns^a^** | **Source** |
| --- | --- | --- | --- | --- |
| Vysis LSI BCL6 Dual Color Break Apart Rearrangement Probe | Break apart | 3’BCL6: green  5’BCL6: red | **Normal Abnormal** | Abbott Molecular |
| Vysis LSI MYC Break Apart Rearrangement Probe Kit |  | 3’MYC: green  5’MYC: red |  |  |
| Vysis LSI BCL2 Break Apart FISH Probe kit |  | 3’BCL2: green  5’BCL2: red |  |  |
| Vysis LSI IGH/MYC/CEP 8 Tri-Color Dual Fusion FISH Probe Kit | Dual fusion | MYC: red  IGH: green  CEP8: blue | **Normal Abnormal** |  |

^a^LSI-Dual-Color-Break Apart Rearrangement Probes: normal nucleus hybridized with these types of probes produces a two orange/green (yellow) fusion pattern. One orange signal, one green signal, and one fusion pattern is expected from a sample having a breakpoint within the gap between the hybridization target genes. The Vysis LSI IGH/MYC/CEP 8 tri Color dual fusion was also studied in both samples. One fusion pattern (yellow), one green signal and one red signal is expected from a case showing an *IGH-MYC* rearrangement. Aqua light identifies two blue signals corresponding to the chromosome 8 centromere.

**Supplementary Table 3.** Morphology, immunophenotype and the multi-step molecular mechanisms implicated in the transformation of FL to a DLBCL is shown from each studied individual biopsy.

**Supplementary Figures:**

**Supplementary Figure 1 .** Conventional cytogenetics:

Conventional cariotyping and FISH studies were done on freshly obtained neoplastic cells from supraclavicular lymphadenectomy (sample A). A complex karyotype was observed: 46,X,-Y,del(3)(p11),+der(8)t(8;?)(p22;?),der(13)t(13;?)(p11;?),der(14)t(14;18)(q32;q21),der(18)t(14;18)(q32;q21)x2[15]. No alterations on chromosome 17 were found.

**Supplementary Figure 2:** FISH studies on lymph node cell culture. Fusion *MYC-IgH* probes for FISH studies identified three copies of *MYC* without rearrangement to *IgH* gene. BA*-break-apart probe. *BCL2, BCL6* and *MYC* studies resulted similar to what was found on paraffin embedded tissue (Figure 1).

**Supplementary Figure 3.** Histological and immunohistochemical studies on Supraclavicular lymph node: Fragments of lymph node diffusely involved by sheets of large neoplastic cells with round or slightly irregular nuclei and a single small nucleolus was seen. Neither necrosis nor vasculitis were found (A and B) (quitar). The cells expressed CD20 (C), BCL2 (D), CD10 (E), MYC (F), BCL6 (G) and p53 (although low, H), and were negative for MUM1, CD30, CD5, TDT, SOX11 and cyclin D1. No EBV (EBER) neoplastic cells were found. The proliferation index (KI67) was moderate to high, but not reaching 90% (I). A diagnosis of GC-DLBCL was made.

**Supplementary Figure 4.** Ileostomy specimen: On histological examination of the intestinal tumor, sheets of medium to large neoplastic cells with round or cleaved nuclei and small nucleoli were found, extending to the perivisceral fat (A,B). These cells expressed CD20, BCL2 (C), CD10 (D), BCL6, MYC (E), p53 and showed a proliferation rate close to 50% (F). No residual CD23/CD21 positive germinal center dendritic cells were identified (G). FISH studies showed *BCL2* and *BCL6* rearrangements (H and I, respectively); with 1F, 1R and 1G dots, in most neoplastic cells, respectively. *MYC* showed 1F and 1R dots in most cells (Figure 1). A diagnosis of HGBC-DH/TH was made.

**Supplementary Figure 5.** Ileostomy specimen: In the adjacent uninvolved mucosa to the neoplastic mass, scattered lymphoid follicles with reactive-looking, secondary germinal centers, were found (A). Higher power view of and slightly abnormal looking germinal center (B). Remarkably, immunohistochemical study highlighted a single germinal center with triple-positive BCL2 (C and D, different increastments)/BCL6/CD10 (F) B-cells, indicative of in-situ follicular neoplasia. *BCL2* study by FISH showed 1F, 1R and 1G dots (E). *MYC* and *BCL6* genes studies by FISH showed two F signals, respectively.

**Supplementary Figure 6**. Ileostomy specimen: Histological study of 20 dissected mesenteric lymph nodes disclosed effacement of their architecture by a partly diffuse, partly nodular proliferation of either small or large cleaved neoplastic cells (A). A higher power-view of large neoplastic cells blurring out the neoplastic follicular centers is seen (B and D). Large cells showed centroblastic morphology (C). Both components expressed CD20 (E), BCL2 (G,H), BCL6 and CD10 (I). There were scattered residual small CD3 positive cells (F). Interestingly, the intensity of staining for BCL2 (G) and CD10 (I) proteins in residual follicules was higher than in the diffusse component. On the contrary, large neoplastic cells of the diffusse component expressed high proliferation index with KI67 (J), MYC (K) and p53 (L). *MYC* and *BCL2* FISH studies showed 1F, 1R and 1G dots. *BCL6* gene showed 2F signals. A diagnosis of DH/HGBCL evolving from a previous follicular lymphoma was rendered.

**References:**

1. Peter Stewart P, Jana Gazdova, MSc, Nikos Darzentas, PhD, Dorte Wren, PhDFRCPath, Paula Proszek, Grazia Fazio, Simona Songia, Miguel Alcoceba, Maria Eugenia Sarasquete, PhD, Patrick Villarese, PhD, Michèle Y van der Klift, Kim Heezen, Neil McCafferty, MSc, Karol Pal, MSc, Mark Catherwood, PhD, Chang Sik Kim, PhD, Shambhavi Srivastava, PhD, Elizabeth Hodges, PhD, Kostas Stamatopoulos, Wolfram Klapper, Simone Ferrero, MD, Michiel van den Brand, MD, Giovanni Cazzaniga, PhD, Frederic Davi, MD PhD, Ramon Garcia-Sanz, MD PhD, Patricia Groenen, Elizabeth Macintyre, PhDMD, Monika Brüggemann, Christiane Pott, MD PhD, Elisa Genuardi, Anton W Langerak, PhD, David Gonzalez, PhD. Euroclonality-NGS DNA Capture Panel for Integrated Analysis of IG/TR Rearrangements, Translocations, Copy Number and Sequence Variation in Lymphoproliferative Disorders. *Blood* 2019; **134** (Supplement_1)**:** 888.
